# Supplementary material for: A solution for 4-propylguaiacol hydrodeoxygenation without ring saturation
Source: Nat Commun. 2024 Jul 27;15:6330. doi: 10.1038/s41467-024-50724-z (PMC11283461; doi:10.1038/s41467-024-50724-z)
Supplement: Supplementary file 1 — Supplementary Info [file 41467_2024_50724_MOESM1_ESM.pdf]

Supporting Information

A Solution for 4-Propylguaiacol Hydrodeoxygenation without Ring Saturation

Zihao Zhang,[1,#] Qiang Li,[2,#] Xiangkun Wu,[1] Claire Bourmaud,[3] Dionisios G. Vlachos,[2,4] Jeremy Luterbacher*,[3] Andras Bodi*,[1] Patrick Hemberger*[1]*

[1] Paul Scherrer Institute, 5232 Villigen, Switzerland

[2] Catalysis Center for Energy Innovation, University of Delaware, 221 Academy St., Newark, Delaware 19716, United States

[3] Laboratory of Sustainable and Catalytic Processing, Institute of Chemical Sciences and Engineering, École Polytechnique Fédérale de Lausanne (EPFL), Station 6, Lausanne, 1015 Switzerland

[4] Department of Chemical and Biomolecular Engineering, University of Delaware, 150 Academy St., Newark, DE 19716, United States

[#] These authors contributed equally

*Corresponding authors. Email: vlachos@udel.edu; jeremy.luterbacher@epfl.ch; andras.bodi@psi.ch; patrick.hemberger@psi.ch

Supplementary Tables

Table S1 Adiabatic ionization energies of various molecules determined with G4 theory.

Name	*m/z*	Chemical Structure	Ionization energy / eV
2-methoxyl-4-propylcyclohexanol	172	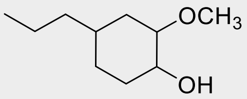	8.65
propylcyclohexane	126	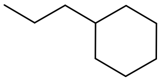	9.47
4-propylphenol	136	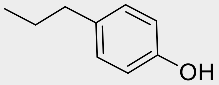	8.05
propylbenzene	120	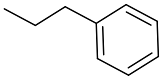	8.71
4-methylcyclohexanol	114	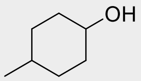	9.61
methylcyclohexane	98	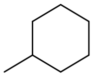	9.69

Table S2 The textural property of the Ru/C catalyst obtained from N_2 adsorption at 77 K.

Sample	Ru/C
BET surface area (m^2/g), (P/P_0 range=0.05~0.2)	671.8
Pore volume (cm^3/g)	0.46
BJH Desorption average pore width (Å)	58.3

Supplementary Figures

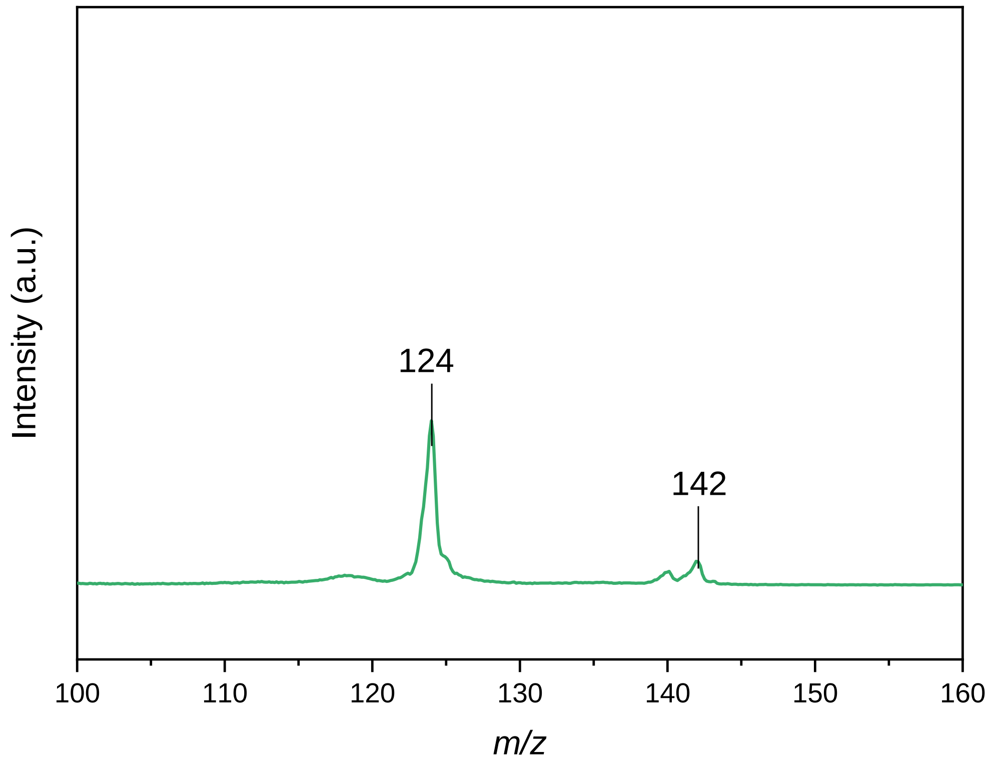

Fig. S1 Photoionization mass spectrum of the reference sample, 4-propylcyclohexanol, at 10.5 eV.

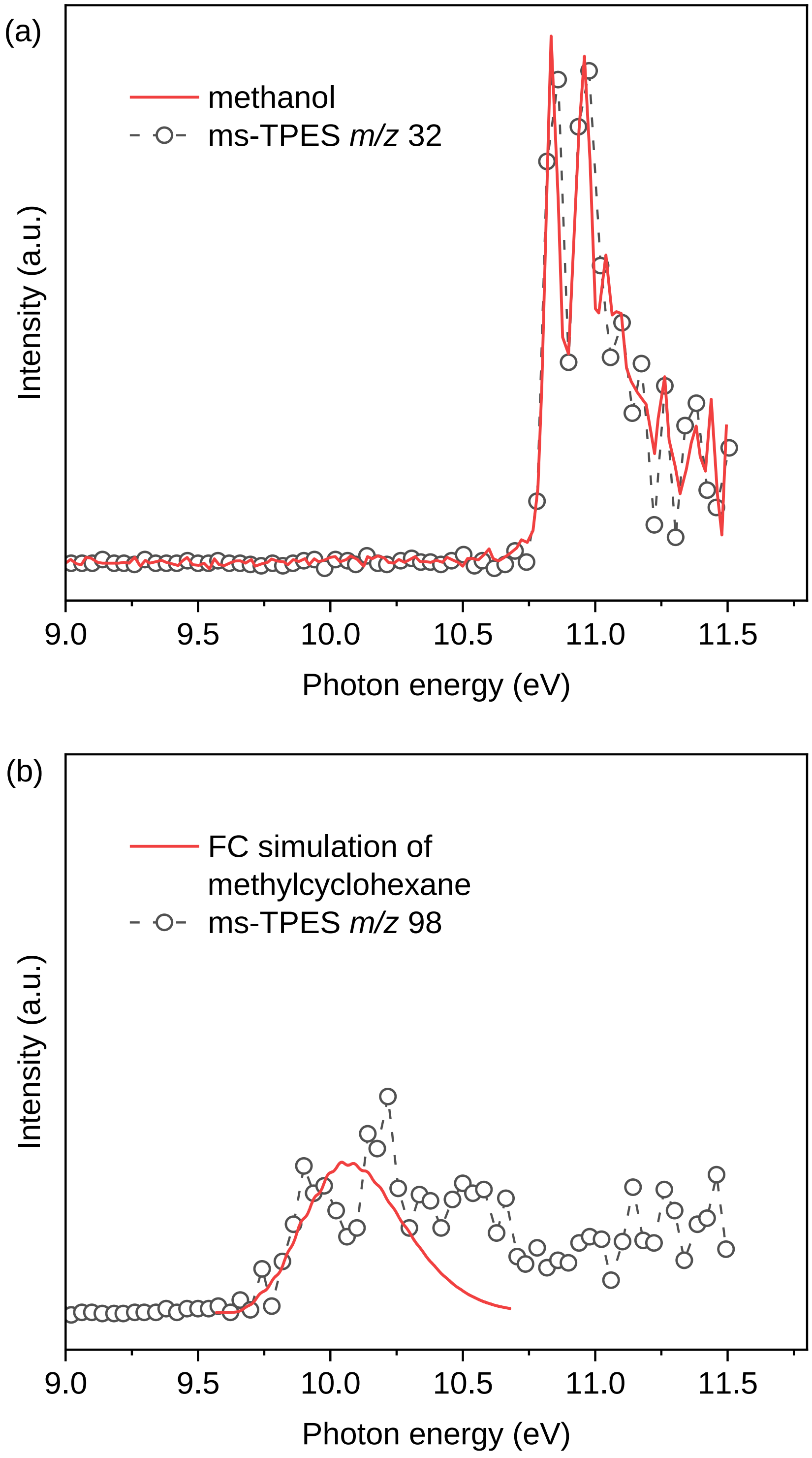

Fig. S2 ms-TPES of (a) *m/z* 32 at 150 °C and (b) *m/z* 98 at 200 °C in 4PG hydrodeoxygenation without solvent. The methanol reference was measured in-house. The methylcyclohexane (MC) reference FC simulation represents the ionization onset due to the ground cation state. Excited states leading to the plateauing ms-TPES intensity are not considered in the simulation.

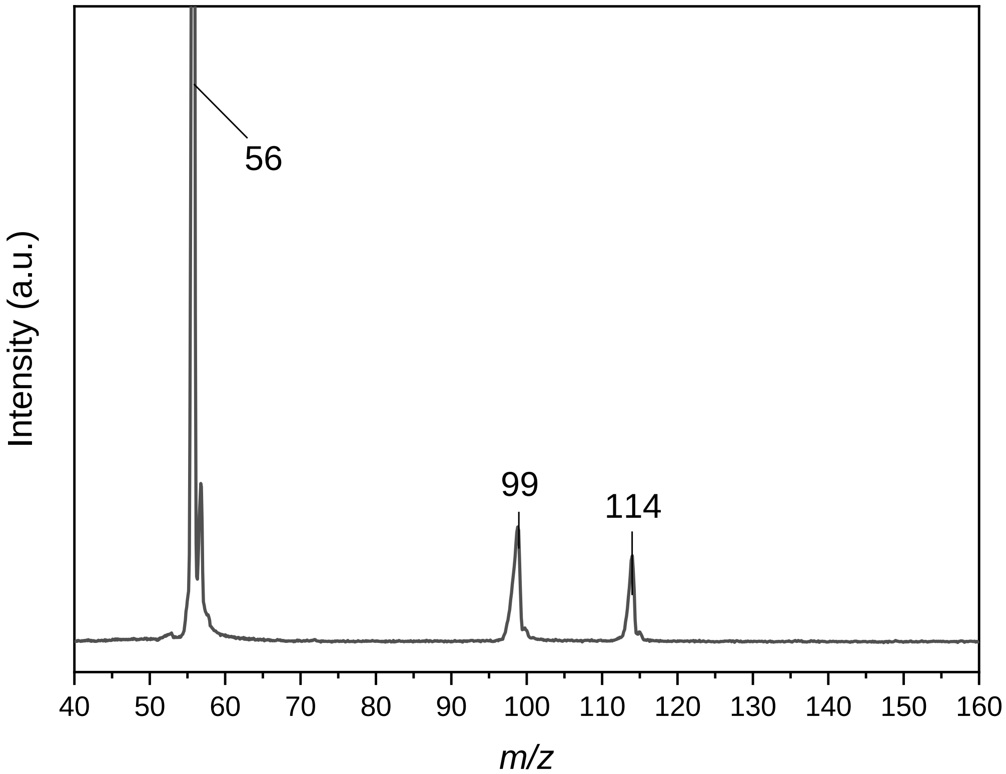

Fig. S3 Photoionization mass spectrum of isooctane at 10.5 eV. In addition to the parent signal at *m/z* 114, dissociative ionization peaks at *m/z* 99, 56 are also detected.

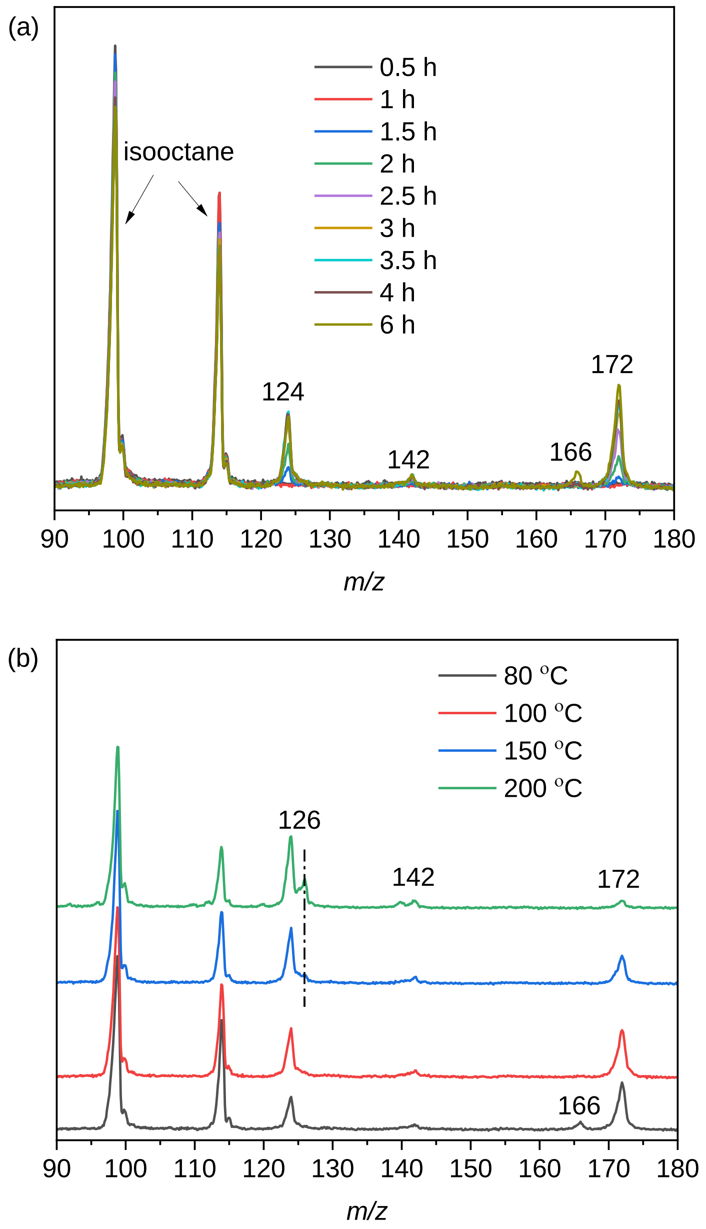

Fig. S4 Photoionization mass spectra for the 4PG hydrodeoxygenation in isooctane at 10.5 eV at (a) 80 °C at different times-on-stream and (b) at different temperatures.

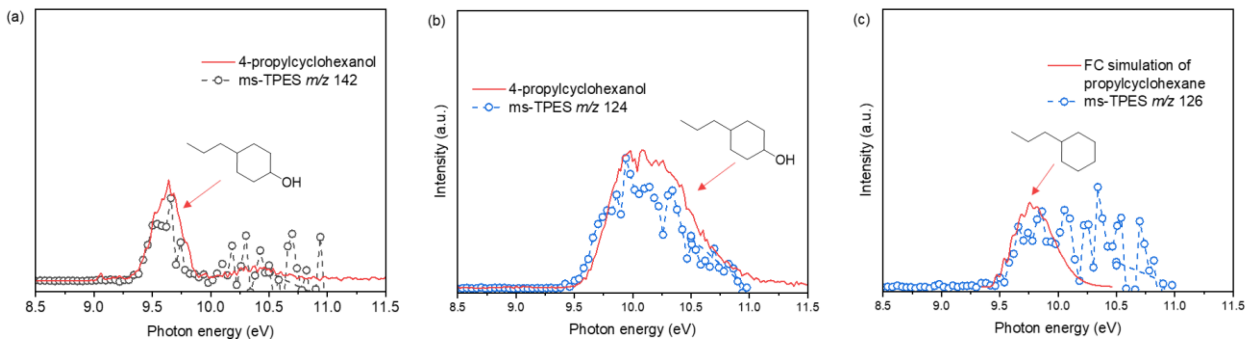

Fig. S5 ms-TPES at 150 °C reactor temperature in the HDO of 4PG in isooctane; (a) *m/z* 142, (b) *m/z* 124, and (c) *m/z* 126.

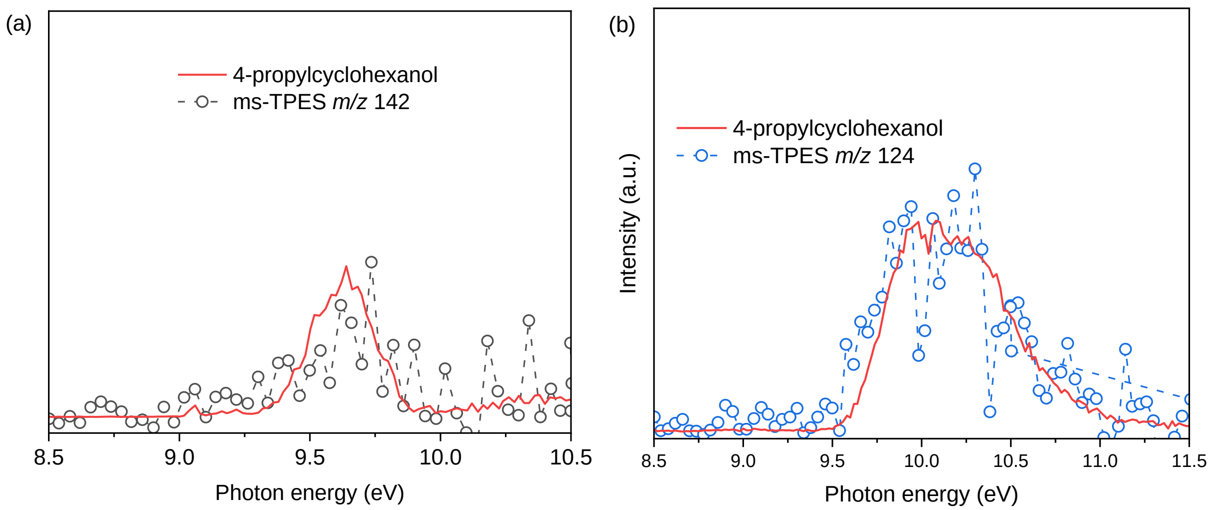

Fig. S6 ms-TPES at 225 °C in 4PG HDO with THF co-feed; (a) *m/z* 142, (b) *m/z* 124.

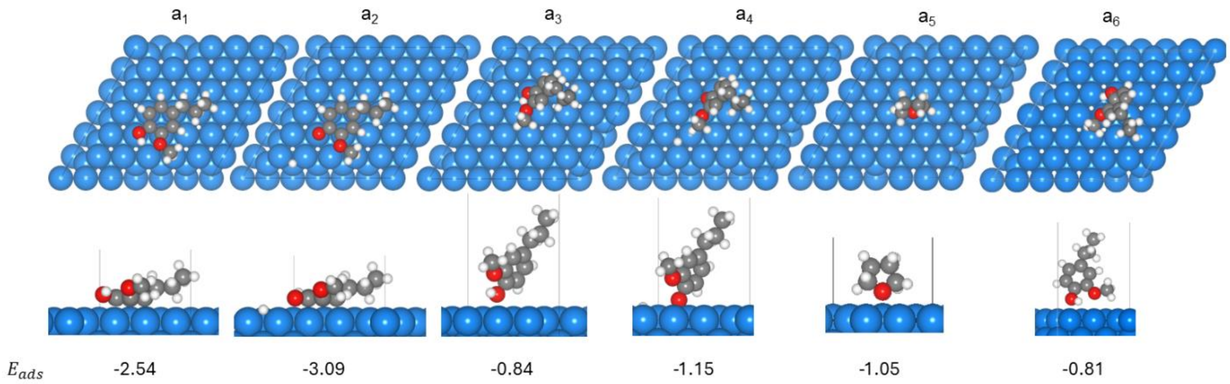

Fig. S7 Top and side view of adsorption configurations of (a_1 – a_4) 4PG and (a_5) THF on the Ru (0001) site. The adsorption energy (*E_{ads}*) is in eV. a_1,a_3: molecular adsorption; a_2,a_4: dissociative adsorption. a_6 represents a 4PG configuration featuring two Ru—O interactions (Ru-OCH_3 and Ru-OH). The structure with two Ru—O interactions show adsorption energy (−0.81 eV) similar to a single Ru—O interaction. This is due to two compensating factors counteracting the increased coordination number: 1) the hydrogen bond between OH and OCH_3 is weakened with respect to a single Ru-O interaction as the length of the hydrogen bond is elongated from 2.018 to 2.172 Å, 2) steric hindrance inhibits the effective interaction between Ru and OCH_3, resulting in a longer Ru—OCH_3 distance (3.035 Å) than in Ru—OH (2.333 Å).

(a)

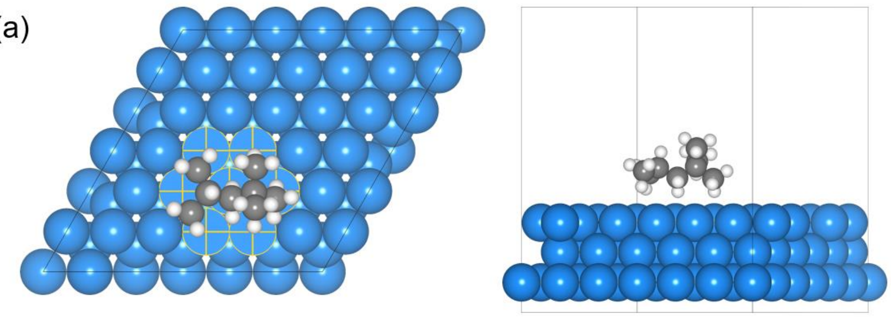

(b)

	Isooctane	THF
$N_{site}^{occupied}$	7	4
E_{ads}	-1.28	-1.05
E_{ads} / site	-0.19	-0.26

Fig. S8 (a) Top and side view of adsorption configurations of isooctane on the Ru(0001) site; (b) the occupied Ru sites and adsorption energy (eV per site) for each configuration for both isooctane and THF.

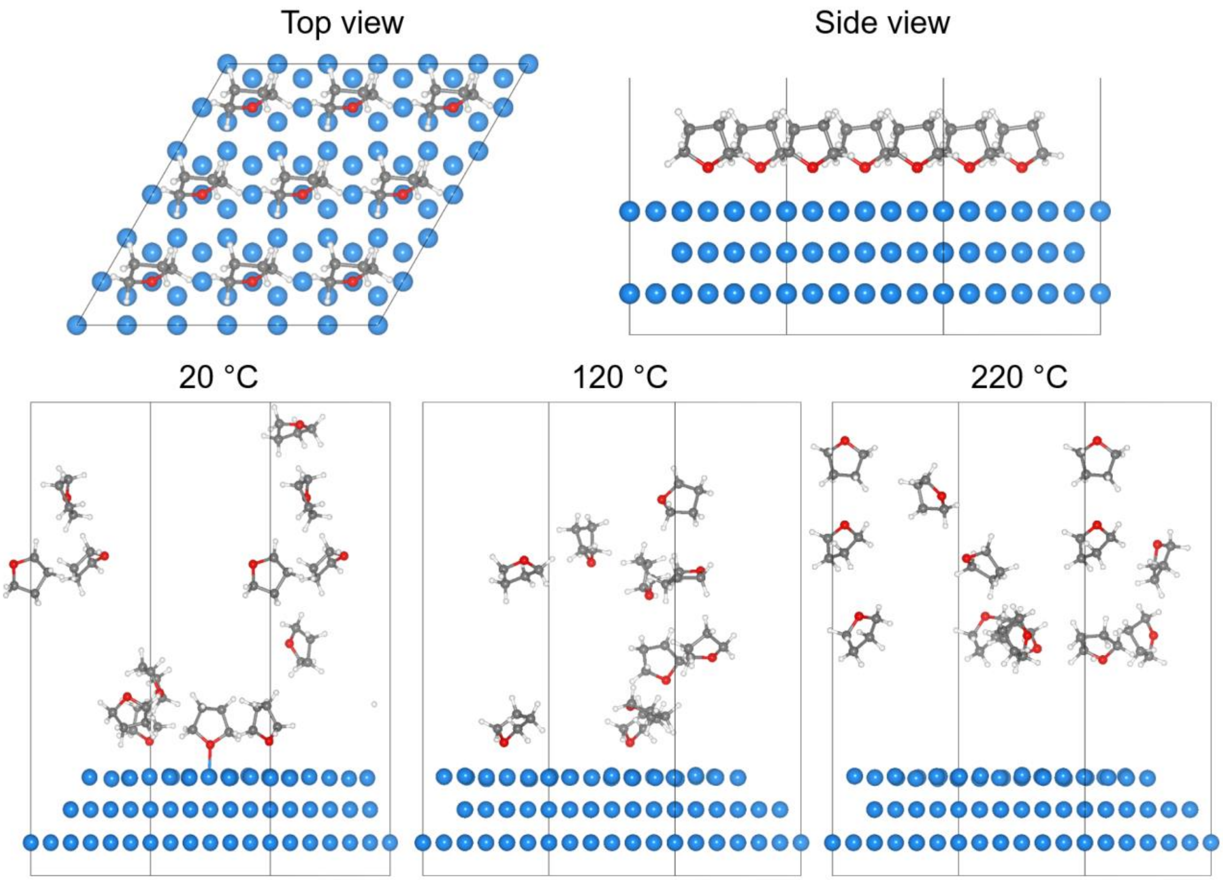

Fig. S9 The top and side view of a configuration with a monolayer THF-covered Ru(0001) surface, as well as MD results at 50 ps at 20, 120, and 220 °C model temperatures.

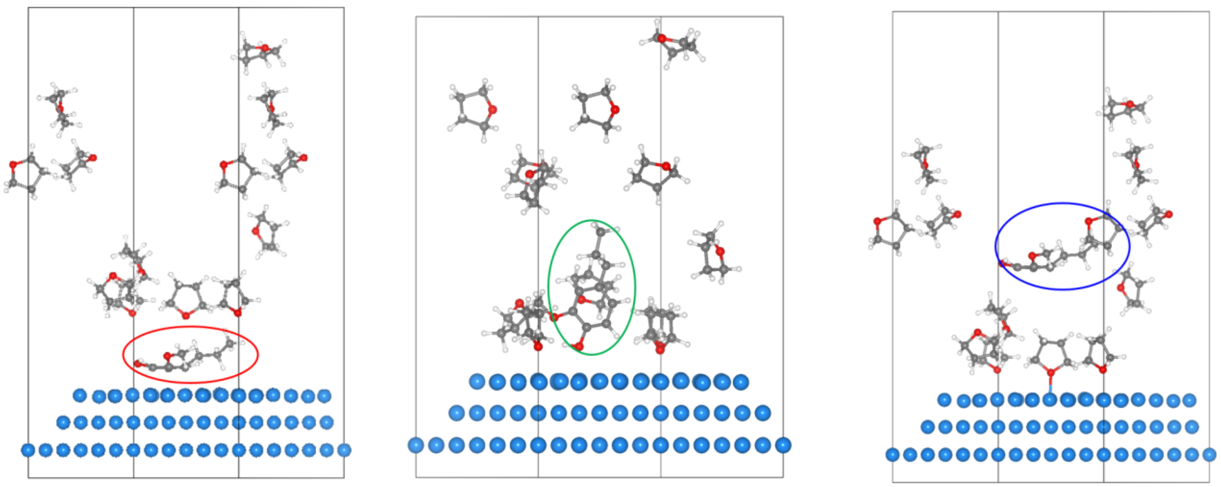

Fig. S10 Initial flat and tilted adsorption configuration of 4PG (circled), as well as the gas-phase 4PG configuration in the presence of THF.

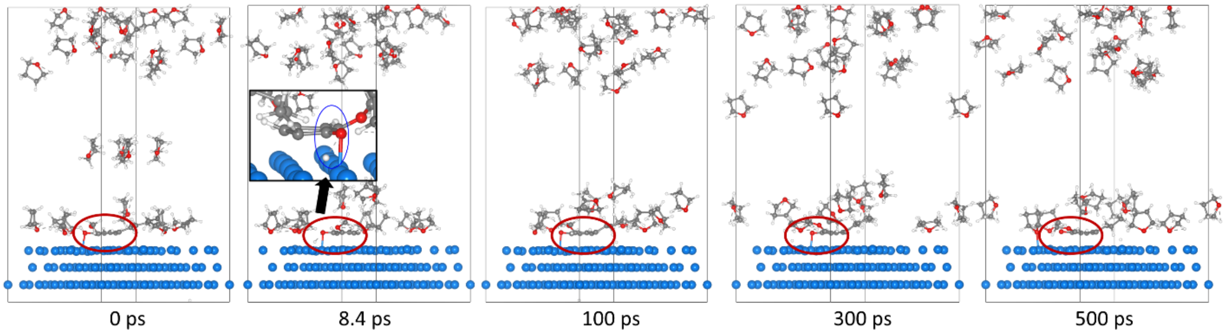

Fig. S11 MLMD starting from flat 4PG (circled) in the presence of THF at 120 °C after 500 ps. The inset at 8.4 ps illustrates the local structure of the O—H bond dissociation.

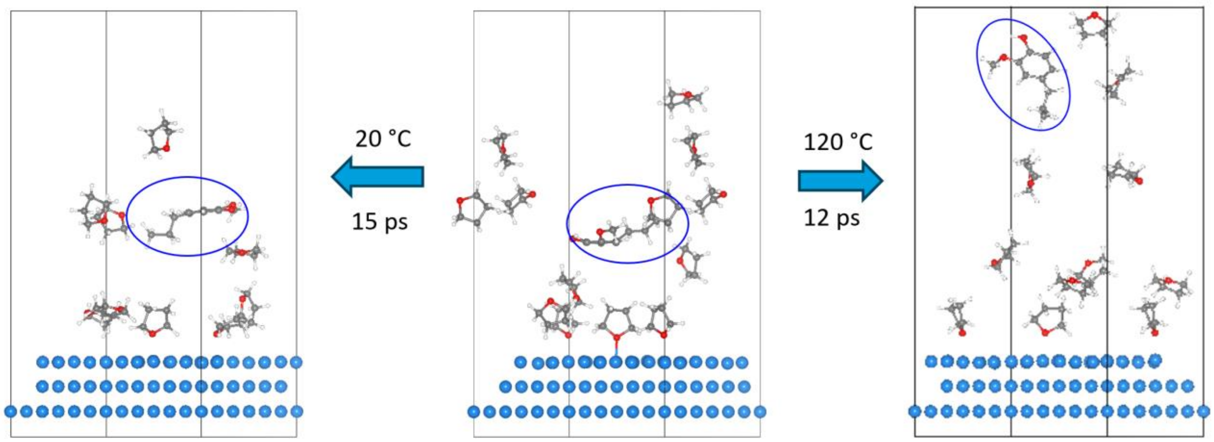

Fig. S12 MLMD starting from gas–phase 4PG (circled) in the presence of THF at 20 °C after 15 ps and at 120 °C after 12 ps.

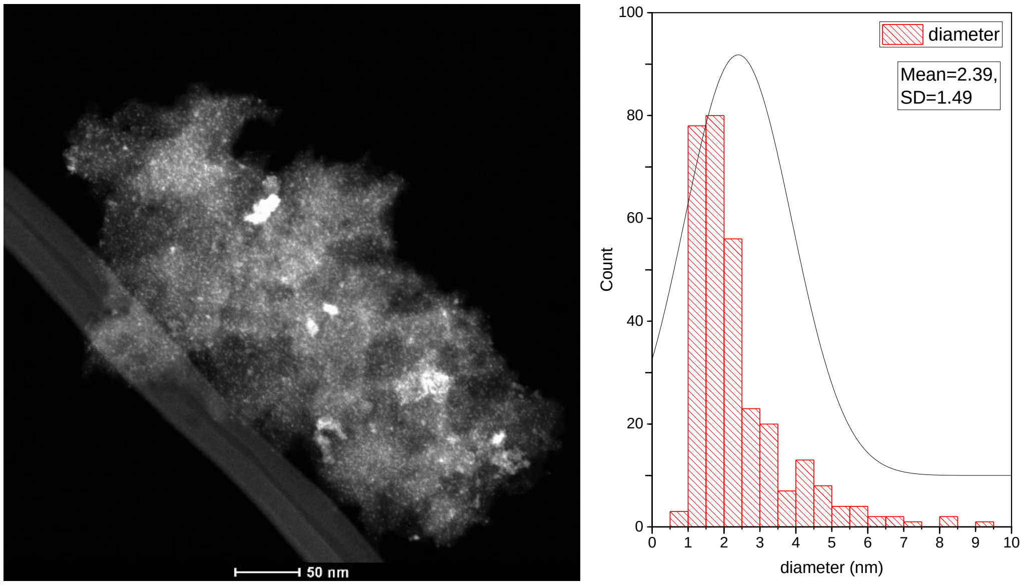

Fig. S13 TEM image of the commercial Ru/C catalyst and its corresponding particle size distribution determined by small cluster analysis. In the TEM image, most Ru particles are highly dispersed with the particle size from 1 to 6 nm. However, the particle size is non-uniform, and there is a small amount of large Ru particles.

On-the-fly MD simulations. On-the-fly MD simulations were performed using VASP versions 6.3 and 6.4. The behaviors of the species on the surface were investigated, and data for force field training was collected from the MD simulations ('ML_MODE = train') of various models containing different numbers of THF molecules and orientations of 4PG. The training set consists of 1600 configurations (different models in a single ML_AB file) with the following corresponding errors (commands: grep BEEF ML_LOGFILE, grep ERR ML_LOGFILE):

BEE energy: 1.17×10^{-6} eV/atom
BEE max force: 0.135 eV/Å
BEE average force: 0.016 eV/Å
RMSE of energies: 1.41×10^{-3} eV/atom
RMSE of forces: 0.096 eV/Å

The force field was refitted for "fast" evaluation using the 'ML_MODE = refit' tag in VASP 6.4 and applied for MD simulations ('ML_MODE = run') over a longer timescale of 500 ps. Following are the Tags in VASP6:

1) One the fly machine learning:
```
#CAL_MD
IBRION = 0
TEBEG = 393
TEEND = 393
MDALGO=2
SMASS = 0
NBLOCK = 10
POTIM = 2
NSW = 5000
ML_LMLFF = .TRUE.
ML_MODE = train
ML_MB = 5000
```
2) Refitting:
```
#CAL_MD
NSW = 1
ML_LMLFF = .TRUE.
ML_MODE = refit
```
3) Prediction
```
#CAL_MD
IBRION = 0
TEBEG = 393
TEEND = 393
MDALGO=2
SMASS = 0
NBLOCK = 100
POTIM = 1
NSW = 500000
ML_LMLFF = .TRUE.
ML_MODE = run
```
